# Expression of 9-*O*- and 7,9-*O*-Acetyl Modified Sialic Acid in Cells and Their Effects on Influenza Viruses

**DOI:** 10.1128/mBio.02490-19

**Published:** 2019-12-03

**Authors:** Karen N. Barnard, Brian R. Wasik, Justin R. LaClair, David W. Buchholz, Wendy S. Weichert, Brynn K. Alford-Lawrence, Hector C. Aguilar, Colin R. Parrish

**Affiliations:** a Baker Institute for Animal Health, Department of Microbiology and Immunology, College of Veterinary Medicine, Cornell University, Ithaca, New York, USA; b Department of Microbiology and Immunology, College of Veterinary Medicine, Cornell University, Ithaca, New York, USA; Virginia Polytechnic Institute and State University

**Keywords:** *O*-acetylation, influenza, sialic acid

## Abstract

Sialic acids (Sia) are widely displayed on the surfaces of cells and tissues. Sia come in a variety of chemically modified forms, including those with acetyl modifications at the C-7, C-8, and C-9 positions. Here, we analyzed the distribution and amounts of these acetyl modifications in different human and canine cells. Since Sia or their variant forms are receptors for influenza A, B, C, and D viruses, we examined the effects of these modifications on virus infections. We confirmed that 9-*O*-acetyl and 7,9-*O*-acetyl modified Sia are widely but variably expressed across cell lines from both humans and canines. Although they were expressed on the cell surfaces of canine MDCK cell lines, they were located primarily within the Golgi compartment of human HEK-293 and A549 cells. The *O*-acetyl modified Sia were expressed at low levels of 1 to 2% of total Sia in these cell lines. We knocked out and overexpressed the sialate *O*-acetyltransferase gene (CasD1) and knocked out the sialate *O*-acetylesterase gene (SIAE) using CRISPR/Cas9 editing. Knocking out CasD1 removed 7,9-*O-* and 9-*O-*acetyl Sia expression, confirming previous reports. However, overexpression of CasD1 and knockout of SIAE gave only modest increases in 9-*O*-acetyl levels in cells and no change in 7,9-*O-*acetyl levels, indicating that there are complex regulations of these modifications. These modifications were essential for influenza C and D infection but had no obvious effect on influenza A and B infection.

## Abstract (precis)

Sialic acids are key glycans that are involved in many different normal cellular functions, as well as being receptors for many pathogens. However, Sia come in diverse chemically modified forms. Here, we examined and manipulated the expression of 7,9-*O*- and 9-*O*-acetyl modified Sia on cells commonly used in influenza virus and other research by engineering the enzymes that produce or remove the acetyl groups.

## INTRODUCTION

**FIG 1:**
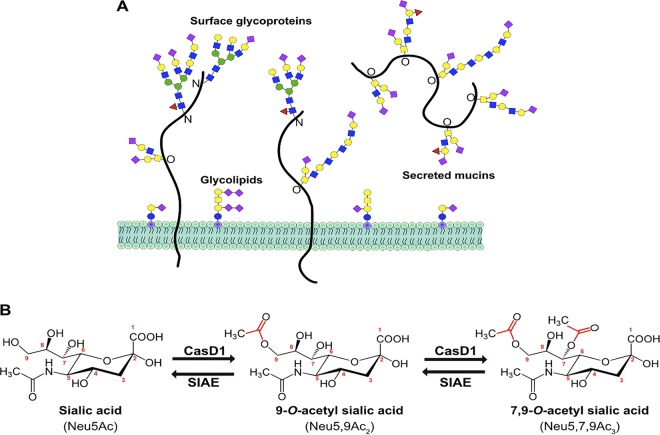
(A) Sialic acids (purple diamonds) terminate glycan chains on glycolipids and glycoproteins as part of the glycocalyx on the surface of cells. They can also terminate glycans on secreted glycoproteins, like mucins, that make up the protective mucosal barrier in gastrointestinal and respiratory tissue. (B) The sialate *O-*acetyltransferase, CasD1, adds acetyl groups to sialic acid (*N*-acetylneuraminic acid [Neu5Ac]) at C-7, from which it migrates to the C-9 position (Neu5,9Ac_2_) under physiological conditions. This can allow for an additional acetyl group to be added by CasD1 to C-7 (Neu5,7,9Ac_3_). The sialate *O-*acetylesterase, SIAE, can remove these acetyl modifications, restoring the unmodified Neu5Ac form of sialic acid.

Sialic acids (Sia) are a family of nine-carbon monosaccharides expressed mainly in vertebrates that serve as terminal residues of carbohydrate chains on cell membrane glycoproteins and glycolipids, as well as on secreted glycoproteins at all mucosal surfaces (Fig. 1A) (1, 2). Sia are key mediators of many cell and tissue functions, where they are bound by cellular receptors such as selectins and siglecs (sialic acid-binding immunoglobulin-type lectins) (3, 4). Their ubiquitous presence on cells, tissues, and mucosal surfaces also make Sia a key point of contact for both commensal microbes and invading pathogens, including viruses, bacteria, and parasites (3).

Sia are highly diverse since there are more than 50 different chemically distinct variants formed from the basic structure of *N*-acetylneuraminic acid (Neu5Ac) by the addition of chemical groups at various positions on the pyranose ring or the glycerol side chain. These modifications include acetyl, sulfo, methyl, and lactyl groups, among others, which may be present individually or in many combinations (1). Since many different enzymes and pathways introduce these modifications, complex mixtures of Sia forms may be present, with significant variation in both the levels and the specific combinations of each modification (1, 2, 5).

Common chemical additions include *O-*acetyl modifications to the C-4, -7, -8, and/or -9 positions, resulting in a variety of combinations, including Neu4,5Ac, Neu5,9Ac_2_, and Neu5,7,9Ac_3_ Sia. The addition of *O-*acetyl modifications (*O*-Ac) to the C-7 and/or C-9 positions is mediated by the sialate *O-*acetyltransferase enzyme, Cas1 domain containing 1 (CasD1) (Fig. 1B). CasD1 appears to add acetyl groups to the C-7 position of Sia, from which it may migrate to the C-8 and C-9 position under physiological conditions, allowing the possible addition of another acetyl group to C-7 (6, 7). A migrase enzyme has been proposed to aid in the transfer of the acetyl group from C-7 to C-9; however, a specific enzyme has yet to be identified (5, 8, 9). CasD1 is localized in the late Golgi compartment, so acetyl modifications are added during the later stages of protein glycosylation. The regulatory processes that control the number of acetyl groups added or their positions have not been well defined, although clear differences in expression of 7,9-*O-*Ac and 9-*O-*Ac have been reported in mouse and human cells and tissues, in chicken embryos, and in the tissues of some other animals (10, 11). CasD1 uses acetyl coenzyme A and likely has a preference for CMP-Neu5Ac as a substrate so that it is less active on CMP-Neu5Gc (7).

At least one sialate *O*-acetylesterase (SIAE) enzyme regulates the display of 7,9-*O-* and 9-*O-*Ac in many cells and tissues (Fig. 1B). The SIAE gene encodes two isoforms that vary in the presence of a proposed C-terminal localization tag in the lysosomal form (Lse) that is absent from the cytosolic form (Cse) (12). However, the processes that regulate the expression and activity of these two isoforms remain poorly defined. Lse has been found to localize to the Golgi compartment and/or endoplasmic reticulum (ER) and on the surfaces of cells when overexpressed, with the majority being secreted into the supernatant (12). Antibody staining shows that Cse is found diffusely throughout the cytosol, where it is thought to remove 9-*O-* and 7-*O*-acetyl groups to recycle the Sia for reuse in glycosylation (12). Despite the reports of distinct protein expression in mouse tissues, bioinformatic analysis of RNA expression in human cells and tissues show mRNA corresponding to the Lse form of SIAE and none responding to the Cse form (13). It is therefore still unclear how these two isoforms are regulated in humans or other animals, whether their expression relates to CasD1, or what controls the levels and locations of 9-*O*- and 7,9-*O*-Ac expression.

Sia *O-*acetylation and deacetylation play important roles in many different biological processes, particularly in development, cancer, and immunology. For example, *O-*acetyl modifications to Sia may alter the binding of host lectins, including siglecs (3, 5, 14). Siglecs are regulators of many different cell functions and developmental processes; examples include B- and T cells, where the presence of 9-*O*-acetyl Sia modulates immune cell activation and differentiation (15). In B cells, negative regulation of B-cell receptor (BCR) activation by siglec CD22 is mediated by binding to Neu5Ac-terminated glycan chains on the BCR, which can be blocked by *O-*acetyl modifications (16, 17). The presence of 9-*O-*Ac can also reduce the activity of sialidases, including human neuraminidases (18). Incorrect regulation of 9-*O-*Ac, 7,9-*O*-Ac, and SIAE activity has been linked to autoimmune disorders through the development of autoantibodies (17, 19). 9-*O-*Ac and 7,9-*O*-Ac, and their regulation by SIAE also appear to play important roles during early stages of embryonic development, spermatogenesis, and in different forms of cancer, including acute lymphoblastic leukemia, colon cancer, and breast cancer (20–23).

Effects of different Sia modifications have also been suggested for the binding of pathogens or on the activities of their sialidases (neuraminidases). However, in general these are still not well documented, with the exception of those that use the modified forms as receptors. Influenza A virus (IAV), IBV, ICV, and IDV use Sia as their primary receptors for host recognition and cell entry, but with different effects of Sia modification. IAV and IBV interact with Sia through two surface glycoproteins, hemagglutinin (HA) and neuraminidase (NA). For both IAV and IBV, HA binds Sia to initiate the endocytic uptake of the virus by the cell, leading to fusion between the viral envelope and the endosomal membrane at low pH (24, 25). NA is a sialidase which cleaves Sia off glycan chains when it is present in mucus or on the surface of cells, allowing the virus to penetrate to the epithelial cells and also preventing aggregation of newly produced virus after budding (26, 27). In contrast to IAV and IBV, ICV and IDV have one surface glycoprotein, the hemagglutinin-esterase fusion protein (HEF), which serves similar purposes to both HA and NA. HEF binds specifically to 9-*O-*acetyl Sia to initiate uptake of the virus into cells, while the esterase domain removes 9-*O-*acetyl modifications, releasing the virus from mucus and disassembling virus aggregates after budding (28–31). While the role of *O-*acetyl modified Sia for ICV and IDV infections are well documented, how these modifications affect IAV is not well characterized. In addition, although previous preliminary studies have suggested that the presence of 9-*O-*Ac on cells may be inhibitory for both NA activity and HA binding of IAV (32, 33), the details are unclear.

Here, we examine and more closely define the expression and distribution of 9-*O*-acetyl and 7,9-*O*-acetyl Sia on cells in culture, define the effects of SIAE and CasD1 on display of these Sia modifications, and perform an initial examination of the effects of these modified Sia on infection by IAV, IBV, ICV, and IDV. We used CRISPR-Cas9 for gene engineering of the enzymes that add or remove the 9-*O-* and 7,9-*O*-acetyl groups from Sia and combine these with the recently developed viral protein-derived probes that specifically recognize modified Sia. By merging these with high-pressure liquid chromatography (HPLC)-based quantification of the different Sia forms, we provide a more detailed understanding of the expression and localization of these modifications in cells and examine their effects on host-virus interactions as examples.

## RESULTS

### Expression of 7,9-*O-* and 9-*O-*acetyl Sia in cells.

**FIG 2:**
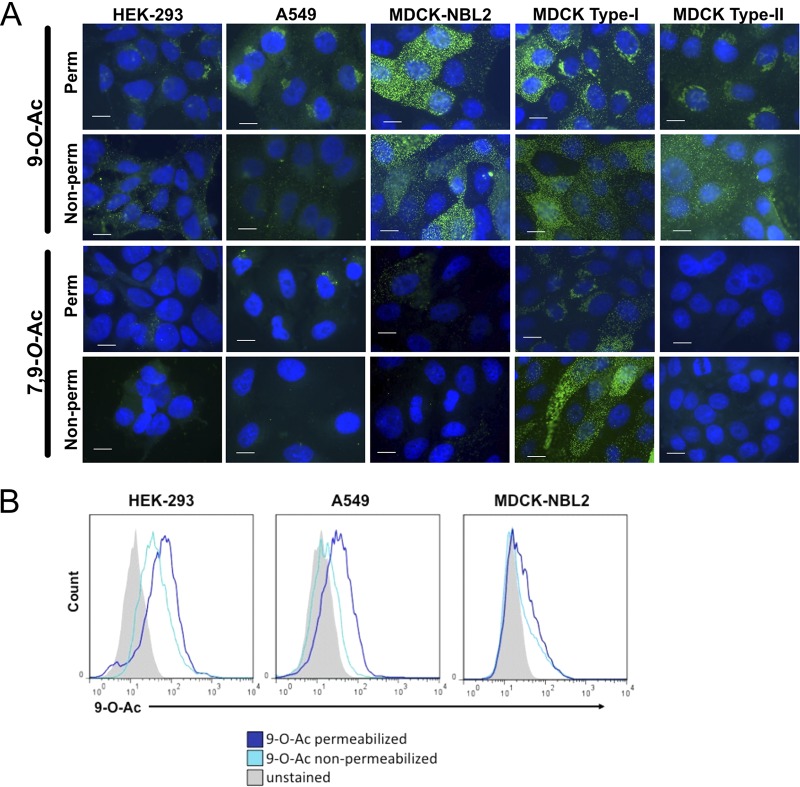
Surface and internal expression of 9-*O*-Ac and 7,9-*O*-Ac on different cell lines. (A) Fluorescent staining of human HEK-293 and A549 and canine MDCK ATCC line (NBL2), MDCK type I, and MDCK type II cells. Cells were probed with HE-Fcs probes derived from BCoV and PToV, which recognize 9-*O*-Ac and 7,9-*O*-Ac, respectively. Cells were permeabilized (perm) using Carbo-Free blocking reagent with 0.001% Tween 20, while nonpermeabilized cells (non-perm) received only Carbo-Free block. All cells were imaged at ×60; nuclei were stained with DAPI. Scale bar, 10 μm. (B) Representative flow cytometry graphs showing distribution of positive staining for HEK-293, A549, and MDCK-NBL2 cell lines. BCoV and PToV HE-Fcs probes were used and permeabilization (perm) and nonpermeabilization (non-perm) methods were as in immunofluorescence assay staining.

**FIG 3:**
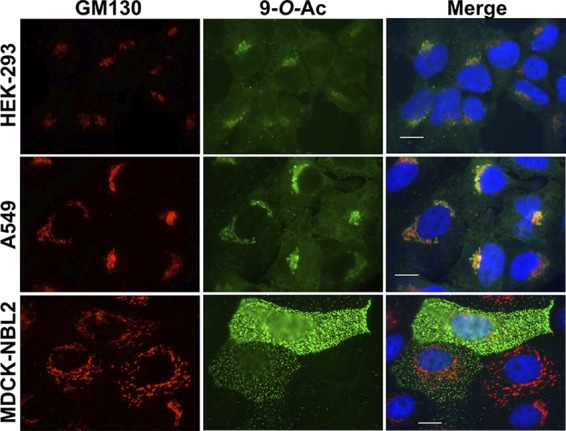
Staining of HEK-293, A549, and MDCK-NBL2 cells with PToV HE-Fc for 9-O-Ac and costaining for the Golgi marker GM130. Cells were permeabilized using 0.001% Tween 20 and imaged at ×60 magnification. Scale bar, 10 μm.

There is currently only sporadic information about the expression of modified Sia on commonly used cell lines or an understanding of how that compares to the expression in animal tissues. We examined cell lines that are widely used in many experimental systems: A549 human type II alveolar epithelial cells, HEK-293 human kidney derived cells (possibly embryonic adrenal precursor cells [34]), and MDCK canine kidney epithelial cells. In addition, we tested MDCK type I and type II cells that were previously subcloned by others from the ATCC MDCK line (MDCK-NBL2) and which have been extensively characterized (35–37). We used probes derived from porcine torovirus (PToV) and bovine coronavirus (BCoV) hemagglutinin-esterase proteins (HE) that were fused to human IgG1 Fc and which had the esterase active site inactivated (HE-Fc). The PToV HE-Fc probe recognizes 9-*O-*Ac, while BCoV HE-Fc recognizes primarily 7,9-*O-*Ac, although with a low affinity for 9-*O*-Ac (10, 11). As observed by immunofluorescence microscopy, the different forms were evident at variable levels, with between 10 and 70% of the cells of each type showing staining under standard culture conditions (Fig. 2). MDCK-NBL2 and MDCK type I cells showed both strong surface and internal staining for 7,9-*O*- and 9-*O*-Ac forms, while both were mostly found in intracellular locations in A549 and HEK-293 cells, with an occasional cell showing bright surface staining. MDCK type II cells showed staining only for 9-*O*-Ac and none for 7,9-*O*-Ac, indicating that these modifications are regulated independently. In HEK-293 and A549 cells, both 7,9-*O*- and 9-*O*-Ac appeared to be localized within the Golgi compartment, as confirmed by costaining with the Golgi marker GM130 (Fig. 3). Similar localization differences between some cell lines have been seen previously using the ICV HEF as a probe (38). In our studies, there was inherent variability within populations in terms of both level of staining and localization. For example, in MDCK-NBL2 not all cells were positive for 9-*O*-Ac, whereas in MDCK type I some cells retained more 9-*O*-Ac and 7,9-*O*-Ac internally, while others displayed more of the modified Sia on their surfaces (Fig. 2A). This heterogeneity was consistent between different passages of each cell line.

The expression levels of those modified Sia variants were also quantified by HPLC analysis using DMB (1,2-diamino-4,5-methylenedioxybenzene) labeling and fluorescence detection (39). The cells were treated at 80˚C for 3 h in the presence of 2 M acetic acid, and therefore the data likely represent the total Sia present in the cells. Even though some cell lines showed strong staining by the HE-Fc probes, the levels of 9-*O-*Ac were 1 to 2% of the total collected Sia from all cells, while the levels of 7,9-*O-*Ac were below the level of detection in all cell lines (Fig. 2 and Table 1 ; see also Fig. S1 in the supplemental material). This includes cells from other species, including cat, mouse, horse, and swine. Unique among the cells tested, A549 cells are secretory and express significant levels of mucin (including MUC1 and MUC5B) (40). Although mucin proteins are not the only secreted protein found in mucus, we chose to focus on them due to their high levels of glycosylation (80% of weight) and proposed importance as a barrier to pathogens (41–43). We found that A549 cells were able to secrete MUC5B into conditioned media (see Fig. S2A in the supplemental material), although we found that the amounts secreted were variable between collections. Analysis of conditioned media from A549 cells showed that approximately 2% of Sia was 9-*O-*Ac on secreted proteins with no 7,9-*O-*Ac detected, indicating that, at least in these cells, secreted proteins were not enriched for *O-*acetylated Sia (Fig. S2B). However, it would be worth examining proteins secreted by primary cells from humans or that are present in different tissues, including mucins, to determine whether the secreted proteins from A549 are representative of respiratory mucus.

**TABLE 1:** Total sialic acids were collected from different cell lines via mild acid hydrolysis and analyzed using HPLC^*a*^

Cell type	Species	Sialic acid content (%)			
		Neu5Gc	Neu5Ac	9-*O-*Ac	7,9-*O-*Ac
A549	Human	2.21	96.40	1.39	ND
HEK-293	Human	2.26	96.72	1.02	ND
MDCK-NBL2	Canine	0.79	97.89	1.32	ND
MDCK type I	Canine	0.66	97.67	1.67	ND
MDCK type II	Canine	0.76	98.13	1.11	ND
NLFK	Cat	2.00	96.87	1.13	ND
EqKc3	Horse	2.93	97.07	ND	ND
NBL6	Horse	2.52	96.38	1.1	ND
L cells	Mouse	3.87	96.13	ND	ND
A72	Canine	2.04	95.85	2.11	ND
SiNEC	Swine*	4.69	93.56	1.75	ND
SiTEC	Swine*	1.97	96.54	1.49	ND

a Percentages of the total sialic acid collected are presented. Representative chromatograms for standard, HEK-293, A549, and MDCK-NBL2 are shown in Fig. S1 in the supplemental material. ND, not detected; *, primary swine nasal (SiNEC) and tracheal (SiTEC) epithelial cells (courtesy of Stacey Schultz-Cherry).

10.1128/mBio.02490-19.1 FIG S1 HPLC chromatograms showing wild-type HEK-293 cells, A549 cells, MDCK-NLB2 cells, and bovine mucin standard of *O-*acetyl modifications. Total sialic acids were collected from cell lines and standards via mild acid hydrolysis. Neu5Gc on cells is likely derived from the fetal bovine serum used in the growth media, which is taken up by cells and displayed on the cell surface. Humans and canines do not have a functional CMAH gene to synthesize Neu5Gc endogenously. Download FIG S1, TIF file, 0.8 MB. Copyright © 2019 Barnard et al. 2019 Barnard et al. This content is distributed under the terms of the Creative Commons Attribution 4.0 International license.

10.1128/mBio.02490-19.2 FIG S2 A549 conditioned medium was analyzed for the presence of secreted mucins and total sialic acid content. (A) Conditioned media from A549 was concentrated using a 30-kDa filter column and then titrated on a western blot for Muc5B expression compared to a purified human Muc5B (a gift from Stefan Ruhl, University of Buffalo). (B) Representative chromatogram of total sialic acid collected from A549 conditioned media using HPLC analysis. The percentage of different sialic acid forms found in total sialic acid collected is summarized in the table. Download FIG S2, TIF file, 0.4 MB. Copyright © 2019 Barnard et al. 2019 Barnard et al. This content is distributed under the terms of the Creative Commons Attribution 4.0 International license.

The low levels of 9-*O-* and 7,9-*O-*Ac detected on HEK-293, A549, and MDCK cells by HPLC could be due to the heterogeneity of the population, since this method measures total Sia for all cells in the population. However, it is likely that even on cells expressing higher levels of 7,9-*O*- and 9-*O*-Ac, these modified Sia make up a small proportion of the total Sia present in the cell or glycocalyx. Previous studies have reported that podoplanin (GP40 in canine cells) is a primary carrier of 9-*O*-Ac on MDCK cells and therefore acts as the main receptor for ICV (44, 45). However, when we costained MDCK cells with an anti-podoplanin antibody and PToV HE-Fc, we saw little correlation between podoplanin and 9-*O*-Ac staining (Fig. S3).

10.1128/mBio.02490-19.3 FIG S3 MDCK-NBL2 cells were costained for the presence of 9-*O-*Ac using PToV HE-Fc (green) and canine podoplanin (red) using an anti-podoplanin antibody (courtesy of Yukinari Kato, Tohoku University). Cells were imaged at ×40 and ×60 magnifications, as indicated. Scale bars, 10 μm. Download FIG S3, TIF file, 1.6 MB. Copyright © 2019 Barnard et al. 2019 Barnard et al. This content is distributed under the terms of the Creative Commons Attribution 4.0 International license.

### Production of CasD1 knockout and CasD1-overexpressing cells.

**FIG 4:**
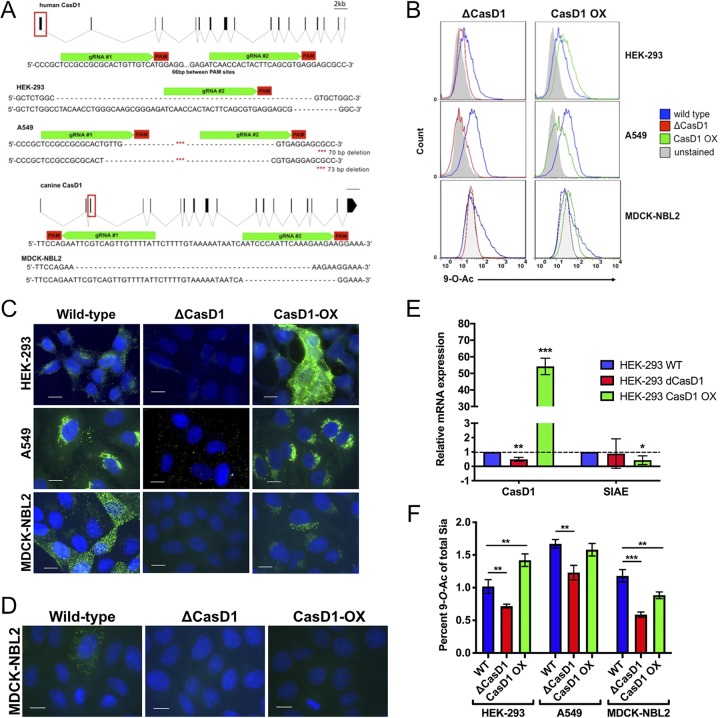
Editing expression of CasD1 in A549, HEK-293, and MDCK-NLB2 cells. (A) Schematic of edits in the CasD1 gene and genotypes of edited cells for HEK-293, A549, and MDCK-NBL2 cells. (B) Phenotype of edited cells by flow cytometry using 9-*O*-Ac probe (PToV HE-Fc). Cells were permeabilized using 0.001% Tween 20 to determine internal expression, since most modified Sia are retained internally. The graph is representative of three independent experiments. (C) Immunofluorescence microscopy images of the different engineered cells stained with PToV HE-Fc to detect 9-*O*-Ac. Cells were permeabilized to reveal both surface and internal expression. Cells were imaged at ×60 magnification. Scale bar, 10 μm. (D) Staining of MDCK WT, ΔCasD1, and CasD1-OX cells showing a representative display of 7,9-*O*-Ac with the BCoV HE-Fc probe. The cells were permeabilized to reveal both surface and internal expression. Cells were imaged at ×60 magnification. Scale bar, 10 μm. (E) qPCR of CasD1 and SIAE expression in HEK-293 WT, ΔCasD1, and CasD1-OX cells. CasD1 still shows mRNA due to a mismatch between qPCR primers and edit sites; mRNA is still present but does not produce functional protein. Expression is indicated relative to the housekeeping gene, GAPDH. Data were analyzed using a *t* test in Prism software. *, *P* ≤ 0.05; **, *P* ≤ 0.01; ***, *P* ≤ 0.001. (F) HPLC data for total Sia collected from cells by mild acid hydrolysis for WT, ΔCasD1, and CasD1-OX cells in HEK-293, A549, and MDCK cells. Data were analyzed by a *t* test using Prism software. *, *P* ≤ 0.05; **, *P* ≤ 0.01; ***, *P* ≤ 0.001.

To better understand the control of expression of 7,9-*O-* and 9-*O-*Ac, glycoengineered cell lines were created by manipulating the expression of CasD1 and SIAE genes. To do this, we knocked out CasD1 via CRISPR-Cas9 editing or overexpressed CasD1 via transfection of an expression plasmid. Knockout variants of CasD1 (ΔCasD1) were prepared from MDCK-NBL2, A549, and HEK-293 cells using CRISPR-Cas9 targeting of early exons in the CasD1 gene (Fig. 4A). ΔCasD1 clones were confirmed by examining for 7,9-*O*- and 9-*O*-Ac display and then by PCR and sequencing of the genomic region surrounding the deletion in modified Sia-negative clones (Fig. 4A). For all cell types, ΔCasD1 variants showed loss of both 7,9*-O*- and 9-*O-*Ac display when probed with the different specific HE-Fc probes via flow cytometry and immunofluorescence microscopy (Fig. 4B to D). This agrees with previous findings that loss of CasD1 leads to loss of both 7,9-*O-* and 9-*O-*Ac modifications in haploid HAP1 cells (7). HPLC analysis of total Sia showed a significant decrease in 9-*O-*Ac expression compared to wild-type (WT) cells; however, very low levels (<1%) were still detectable, despite a lack of staining with the HE-Fc probes (Fig. 4E). This could be due to exogenous Sia from the fetal bovine serum in the growth media, as is the case for Neu5Gc, which is also detectable at similarly low levels on cells even though the gene for Neu5Gc synthesis, CMAH, is not functional in humans or canines (Fig. S1) (46, 47).

Due to the heterogeneity and low expression of 7,9-*O*- and 9-*O*-Ac in WT cells, we sought to engineer cells with more homogeneous and higher levels of these modifications. We overexpressed the human CasD1 (CasD1-OX) in the ΔCasD1 variants of MDCK-NBL2, A549, and HEK-293 cells, expecting that expression of CasD1 under a strong promoter in the ΔCasD1 background would increase the consistency of the synthesis and display of 9-*O*- and 7,9-*O*-acetyl Sia relative to the WT cells. CasD1-OX cells showed significantly higher levels of CasD1 mRNA compared to WT cells, indicating that the CasD1 expression plasmid was being transcribed at high levels (Fig. 4E). However, only a modest increase in average 9-*O*- and 7,9-*O*-Ac expression across the population in HEK-293 cells was seen by flow cytometry, whereas fluorescence microscopy showed heterogeneity of expression in the population (Fig. 4B to D). The transfected MDCK and A549 CasD1-OX cells showed recovery of 9-*O*- and 7,9-*O*-Ac synthesis when analyzed by flow cytometry and immunofluorescence microscopy, but the levels were not as high as those seen in WT cells (Fig. 4B to D). HPLC analysis confirmed the expression levels for HEK-293, A549, and MDCK cells (Fig. 4F). In addition, heterogeneity was still seen in these populations for 9-*O*-Ac. This suggests that 7,9-*O-* and 9-*O-*Ac expression is not directly regulated by the levels of CasD1 gene expression but may be affected by posttranslational regulation of CasD1 activity or removal of the modifications by SIAE or other enzymes. In addition, these modifications are not regulated the same across individual cells, as evidenced by the population heterogeneity. SIAE transcripts were consistently expressed in HEK-293 WT, ΔCasD1, and CasD1-OX cell populations by qPCR analysis, although SIAE mRNA levels in CasD1-OX cells were lower than those seen in WT (Fig. 4E).

### SIAE knockout cells and display of modified Sia.

**FIG 5:**
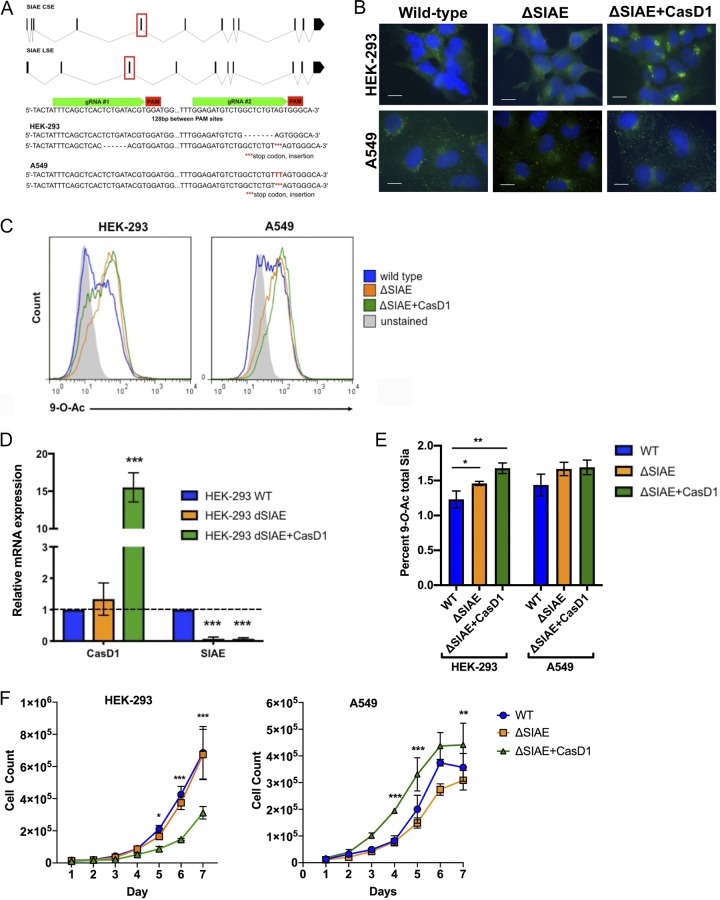
Editing the expression of SIAE in HEK-293 and A549 cells. (A) Schematic of edits in the SIAE gene, which was targeted to remove both isotypes of SIAE. The genotypes of edited cells show frameshifts that lead to stop codons in all cases. (B) Staining of the different engineered cells with PToV HE-Fc to detect 9-*O*-Ac. Cells were permeabilized using 0.001% Tween 20 to determine surface and internal expression. Cells imaged at ×60. Scale bar, 10 μm. (C) Flow cytometry using PToV HE-Fc showing the relative display of 9-*O-*Ac. Cells were permeabilized using 0.001% Tween 20 to show both surface and internal expression. The graph is representative of three independent experiments. (D) qPCR of relative SIAE and CasD1 mRNA expression in HEK-293 WT, ΔSIAE, and ΔSIAE+CasD1 cells compared to GAPDH. SIAE qPCR primers overlap with edit site. Data were analyzed by a *t* test using Prism software. *, *P* ≤ 0.05; **, *P* ≤ 0.01; ***, *P* ≤ 0.001. (E) HPLC data for total Sia collected from cells by mild acid hydrolysis for WT, ΔSIAE, and ΔSIAE+CasD1 cells in HEK-293 and A549 cells. Data were analyzed by a *t* test using Prism software. *, *P* ≤ 0.05; **, *P* ≤ 0.01; ***, *P* ≤ 0.001. (F) Growth curve for WT, ΔSIAE, and ΔSIAE+CasD1 cells in HEK-293 and A549 cells. Cells were counted every 24 h. Each experiment was performed in triplicate. Data were analyzed by two-way analysis of variance (ANOVA) using Prism software. *, *P* ≤ 0.05; **, *P* ≤ 0.01; ***, *P* ≤ 0.001.

To determine the role of SIAE activity in regulating 7,9-*O*- and 9-*O*-Ac display, SIAE knockout (ΔSIAE) cells were generated from WT HEK-293 and A549 cells by targeting early exons in the SIAE gene (Fig. 5A). Complete knockout of SIAE was confirmed both by genotyping to show deletions in both alleles and by loss of mRNA by qPCR (Fig. 5A and D). ΔSIAE cells showed a small but significant increase in surface display and a large increase in internal display of 9-*O-*Ac based on flow cytometry, but they did not appear to show any changes in the surface or internal display of 7,9-*O-*Ac (Fig. 5B and C). HPLC analysis also showed a small increase of 9-*O*-Ac in HEK-293 cells, but no 7,9-*O*-Ac was detected in either cell line (Fig. 5E). When the overexpression CasD1 plasmid was transfected into ΔSIAE cells (ΔSIAE+CasD1), the cells showed an increase in both surface and internal 9-*O*-Ac but no increase in surface or internal display of 7,9-*O*-Ac by either flow cytometry or immunofluorescence microscopy (Fig. 5B and C). HPLC confirmed the flow cytometry results by showing a small increase of 9-*O*-Ac levels in HEK-293 cells, similar to those seen in CasD1-OX (Fig. 5E). A549 cells showed a small but nonsignificant increase in 9-*O-*Ac levels compared to WT by HPLC analysis. Interestingly, HEK-293 ΔSIAE+CasD1 cells grew more slowly than either ΔSIAE or WT cells (Fig. 5F). However, A549 ΔSIAE+CasD1 showed increased growth rates compared to ΔSIAE or WT cells, while ΔSIAE cells had slightly lower growth rates compared to WT. This suggests that 7,9-*O-* and 9-*O*-Ac may affect cell metabolism and growth rates and that these effects may be cell type specific. Overall, these results show that SIAE regulates levels of 9-*O*-Ac and 7,9-*O*-Ac but that knocking out SIAE only leads to small increases in these modifications. However, there appear to be mechanisms regulating the surface display of 9-*O*-Ac and 7,9-*O*-Ac, since most of the modified Sia were specifically retained in the Golgi compartment on human HEK-293 and A549 cells, in comparison to WT MDCK cells, which appear to display most modified Sia on their surfaces.

### Effects of 7,9-*O*-Ac and 9-*O*-Ac on influenza A, B, C, and D virus infection.

**FIG 6:**
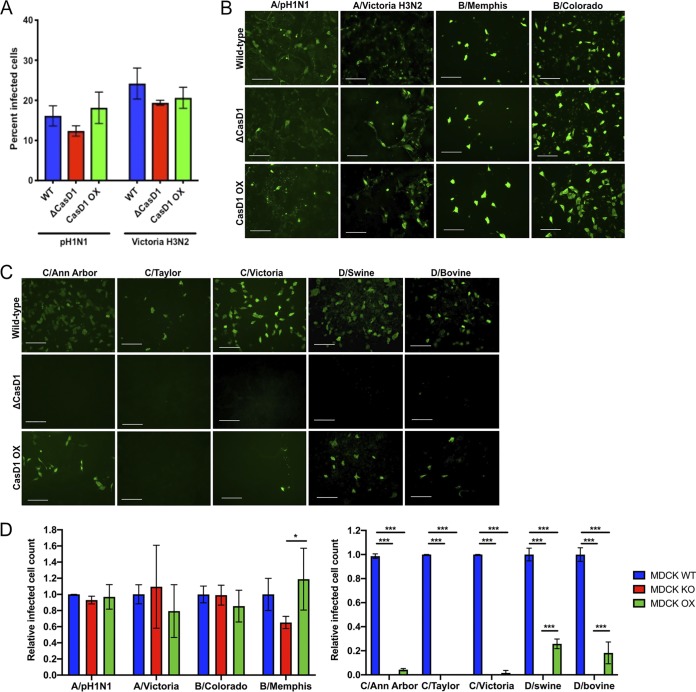
Infection of WT, ΔCasD1, and CasD1-OX cells with IAV, IBV, ICV, and IDV. (A) HEK-293 WT, ΔCasD1, CasD1-OX cells were inoculated at an MOI of 0.1 with IAV strains pH1N1 (A/California/04/2009) and Victoria H3N2 (A/Victoria/361/2011). The cells were fixed at 24 h, and the infected cells per field were counted. Each experiment was performed in triplicate. Data analyzed by two-way ANOVA using Prism software. (B) MDCK WT, ΔCasD1, and CasD1-OX cells were inoculated at a high MOI with IAV strains pH1N1 (A/California/04/2009) and Victoria H3N2 (A/Victoria/361/2011, and IBV strains B/Memphis/1/2018 and B/Colorado/06/2017 for 48 h and then imaged at ×10 magnification. Scale bar, 100 μm. Representative images of three independent experiments are shown. (C) MDCK WT, ΔCasD1, and CasD1-OX cells were inoculated at a high MOI with ICV strains C/Ann Arbor/1/50, C/Taylor/1233/1947, and C/Victoria/1/2011 and IDV strains D/bovine/MS/C000020N/2014 and D/swine/OK/1334/2011 for 48 h and then imaged at ×10 magnification. Scale bar, 100 μm. Representative images of three independent experiments are shown. (D) Quantification of the relative infected cell counts for panels B and C, as determined through image analysis with ImageJ. Data were analyzed by two-way ANOVA using Prism software. *, *P* ≤ 0.05; **, *P* ≤ 0.01; ***, *P* ≤ 0.001.

To test the effects of 9-*O*-Ac and 7,9-*O*-Ac on virus infection, WT, ΔCasD1, and CasD1-OX HEK-293 cells were inoculated with human H1N1 (A/California/04/2009) and human H3N2 (A/Victoria/361/2011) IAV strains, and we found no significant difference in infection efficiency in any of these cells (Fig. 6A). IAV strains, as well as IBV strains B/Colorado/06/2017 and B/Memphis/1/2018, also showed equal infection efficiency on WT MDCK cells compared to ΔCasD1 and CasD1-OX cells (Fig. 6B and D). B/Memphis did show a difference in relative infected cell counts between ΔCasD1 and CasD1-OX, although B/Colorado did not. However, ICV strains C/Ann Arbor/1/50, C/Taylor/1233/1947, and C/Victoria/1/2011, as well as IDV strains D/bovine/MS/C00020N/2014 and D/swine/OK/1334/2011, showed no infectivity in MDCK ΔCasD1 cells (Fig. 6C and D). The C/Ann Arbor and C/Victoria recovered a low level of infectivity in the MDCK CasD1-OX cells, whereas the D/bovine and D/swine strains had a higher level of infectivity recovered in the CasD1-OX cells compared to ICV but still were much lower than in WT MDCK cells. The inability of the ICV and IDV strains to fully recover infectivity may be due to the low surface levels of 9-*O*-Ac Sia on MDCK CasD1-OX cells. Although the CasD1-OX cells had detectable surface levels of 9-*O-*Ac, these levels were lower than in WT MDCK cells and could be below the receptor levels required for efficient infection by these viruses. This suggests that ICV and IDV are able to utilize the levels of modified Sia on WT MDCK cells as their primary receptors for binding and infection but cannot infect when they are removed or below a certain necessary threshold.

### Effects of 7,9-*O*- or 9-*O*-acetyl Sia modifications on HA binding and NA activity.

**FIG 7:**
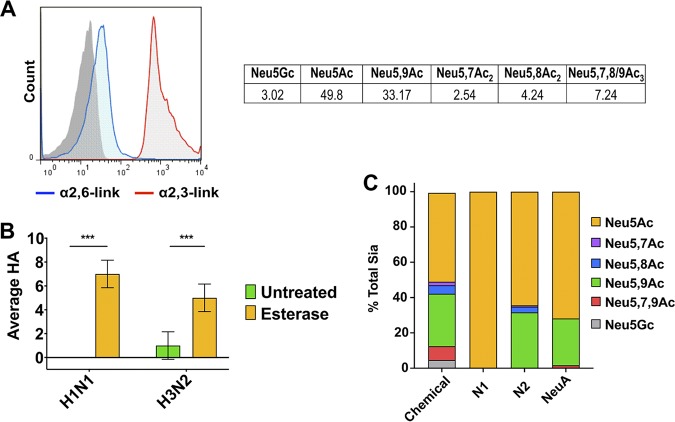
Effects of 7,9-*O*- and 9-*O-*Ac on IAV HA binding and NA cleavage. (A) Surface staining for α2,6-linked Sia and α2,3-linked Sia on mouse erythrocytes via flow cytometry and a table showing total Sia analysis of mouse erythrocytes using HPLC. Data for each Sia variant are given as the percentages of total Sia, averaged across three independent samples. (B) Hemagglutination of human H1N1 and H3N2 IAV strains on untreated or esterase-treated mouse erythrocytes. The data are averaged across three independent experiments, with data analyzed by two-way ANOVA using Prism software. (C) Profiles of total Sia freed by either chemical treatment, N1 VLPs, N2 VLPs, or commercial NeuA sialidase as determined by HPLC. The data are averaged across two independent experiments.

Since the low levels of 7,9-*O-* and 9-*O-*AC on the surfaces of cells did not effect IAV infection in our assays, we sought to determine the effects of these modifications on HA binding and NA cleavage using mouse erythrocytes which have ∼45% of their total Sia is *O-*acetylated, primarily 7,9-*O*- and 9-*O-*Ac (Fig. 7A). Although mouse erythrocytes contain primarily α2,3-linked Sia, they also contain some α2,6-linked Sia that could be bound by human IAV strains (Fig. 7A). Mouse erythrocytes were therefore used as a substrate for both binding (hemagglutination) assays and neuraminidase cleavage assays. The human IAV strains human H1N1 (A/California/04/2009) and human H3N2 (A/Victoria/361/2011) strains were mixed with either untreated mouse erythrocytes or mouse erythrocytes pretreated with esterase active BCV HE-Fc to remove all acetyl modifications (Fig. 7B). Significantly greater hemagglutination was seen for both viruses on the esterase-treated mouse erythrocytes compared to untreated erythrocytes. It should be noted that the hemagglutination was still quite low, which is likely due to the lower levels of α2,6-linked Sia on mouse erythrocytes.

To determine the effect of 7,9-*O*- and 9-*O-*Ac on NA cleavage, mouse erythrocytes were incubated with soluble NA, in the form of NA-expressing viruslike particles (VLPs) (48). Freed Sia was then collected and analyzed using HPLC, and the profile of Sia released by the NA VLPs was compared to Sia composition on mouse erythrocytes released by chemical hydrolysis (Fig. 7C). The profiles for both N1 and N2 VLPs showed a preferential release of unmodified Neu5Ac compared to modified Sia forms, shown in the increased proportion of this Sia in the N1 and N2 profiles. Compared to the chemical release profile, the N1 VLPs did not release any detectable *O-*acetylated Sia, while the N2 VLPs were able to release 9-*O-*Ac (Neu5,9Ac_2_) but released significantly less 7-*O-* and 8-*O-*Ac and were unable to release any 7,9-*O-*Ac (Neu5,7,9Ac_3_). Similar to N2 VLPs, commercial NeuA from Arthrobacter ureafaciens, used as an activity control, also had a bias toward unmodified Neu5Ac, with decreased activity against 7-*O-*, 8-*O-*, and 7,9-*O-*Ac. These results indicate that mono *O*-acetyl modifications, including 7-*O-*, 8-*O-*, and 9-*O-*Ac, reduced the Sia susceptibility to NA cleavage and that diacetyl 7,9-*O-*Ac was the most resistant to NA cleavage. In addition, there was variability between the ability of the NA VLPs and NeuA to cleave the different Sia forms. Although the presence of these modified Sia on cells was too low to affect IAV infection, it is likely that higher levels present on erythrocytes, or potentially in secreted proteins in mucus, would inhibit infection or the release of virus.

## DISCUSSION

Both 9-*O*-Ac and 7,9-*O*-Ac Sia are widely expressed within tissues and on mucosal surfaces of many animals, but with significant variation in the amounts present in different cells, tissues, and animals (10, 11). These modified Sia are present in secreted mucus on mucosal surfaces, including gastrointestinal and respiratory tissues, where they can potentially control many interactions with both normal flora and pathogens. However, there is still little known about the details of their display levels, cell association, and the ways in which their synthesis is regulated either in cells or on mucosal surfaces. While there have been suggestions that 9-*O*- and 7,9-*O*-Ac might influence IAV and IBV infection by interfering with HA binding or NA activities, direct evidence for their effects is sparse. In contrast, ICV and IDV are known to use 9-*O*-Ac as their primary receptor for cell binding and infection. Here, we use a number of new tools to define the cell-specific expression of 9-*O*- and 7,9-*O*-Ac and provide a preliminary test of their effects on IAV, IBV, ICV, and IDV infection.

**FIG 8:**
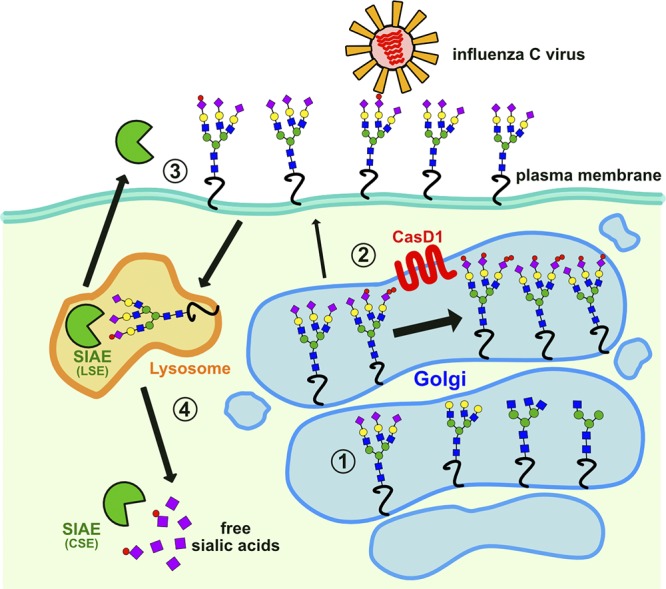
Summary of proposed 7,9-*O-* and 9-*O-*acetyl sialic acid production and trafficking in cells. (Step 1) Sia (purple diamond) is added to the growing glycan chain in the Golgi by sialyltransferases using CMP-Neu5Ac or CMP-Neu5Gc substrates, which are synthesized in the nucleus by the addition of cytodine monophosphate (CMP) to Neu5Ac or Neu5Gc and are specifically imported into the Golgi. (Step 2) CMP-Neu5Ac or CMP-Neu5Gc Sia are modified by CasD1, adding one or two acetyl groups to form 9-*O-*Ac or 7,9-*O-*Ac, respectively (red circles), before being added to glycan chains. The majority of glycoproteins with these modifications are retained in the Golgi compartment (large arrow) of many cells, including the HEK-293 and A549 cells examined here, whereas only some (mostly 9-*O-*Ac) are transported to the cell surface (small arrow). (Step 3) Surface-displayed *O-*acetyl Sia can interact with pathogens, cell receptors, or lectins. For example, ICV uses 9-*O-*Ac as its receptor. Secreted forms of SIAE may also remove the *O-*acetyl modifications, altering these lectin-ligand interactions. (Step 4) When glycoproteins are recycled from the cell surface, the lysosomal form of SIAE (LSE) can remove *O-*acetyl modifications from Sia. Free Sia are exported to the cytosol, where the cytosolic form of SIAE (CSE) can also remove any remaining *O-*acetyl modifications. Unmodified Sia can then be “activated” in the nucleus by the addition of CMP and transported to the Golgi compartment for addition to new glycan chains.

We confirmed that 9-*O*- and 7,9-*O*-Ac are expressed on cells in culture and that expression varies between cell lines, as has been previously reported (38). However, when present these modified Sia made up only 1 to 2% of the total Sia when analyzed by HPLC. Cells showed distinct population heterogeneity in 7,9-O- and 9*-O-*Ac display and localization that was observed over many passages examined. Previous studies using ICV HEF probes found similar staining patterns on some cell lines and also showed that, after sorting into high and low staining populations, both populations returned to previous levels of heterogeneity within a few passages (11, 38). Here, we saw that the modified Sia are retained in the Golgi compartments of HEK-293 and A549 cells, although an occasional cell displayed these modified Sia on the surface. It is not clear why these modified Sia are localized in the Golgi body, although perhaps the modifications could block the onward trafficking of glycoproteins to the cell surface. In contrast to the human cell lines, MDCK cells showed many cells expressing 7,9-*O*-Ac and 9*-O-*Ac on the cell surface, raising the possibility that trafficking could be cell type or species specific. A summary of the synthesis and localization of 7,9*-O-* and 9-*O-*Ac is shown in Fig. 8.

To look more closely at the expression and roles of 7,9-*O*- and 9-*O*-Ac modifications and their effects on viral infections, we prepared glycoengineered cell lines that lacked these modifications or that expressed higher levels of CasD1. A deletion and frameshift in CasD1 completely removed both 9-*O*- and 7,9-*O*-Ac expression, confirming this enzyme was responsible for creating both modifications, likely through addition to the C-7 position, from which it migrates to the C-9 position (5, 8, 9). Adding CasD1 back into the cells by plasmid transfection restored modified Sia expression, but none of the cell clones isolated showed universally higher levels of modified Sia synthesis and population heterogeneity was still seen. The CasD1-transfected HEK-293 cells showed the greatest increase, whereas for A549 and MDCK cells the expression was similar to or lower than that for WT cells. However, even in the HEK-293 CasD1-OX cells, 9*-O-*Ac still only accounted for ∼1.5% of total Sia, and 7,9-O-Ac was not detected. This indicates that the levels of these modifications are not only controlled by the expression of CasD1 and could be regulated by other processes. In addition, there is clearly a differential regulation of 7,9-*O-*Ac compared to 9-*O-*Ac, as overexpressing CasD1 did not lead to an increase in 7,9-*O-*Ac, since it was not detectable by HPLC and there was only very low fluorescence via staining with BCoV HE-Fc.

One candidate for control of these modifications is SIAE. When we knocked SIAE out of HEK-293 and A549 cells, we saw an increase in 9-*O*-Ac that was still retained in the Golgi compartment, but there was no increase in 7,9-*O*-Ac. Expressing CasD1 from a plasmid in ΔSIAE HEK-293 and A549 cells resulted in an additional small increase in 9*-O-*Ac that was still Golgi compartment associated, but with little increase in expression on the cell surface. Similar to the CasD1-OX cells, no increase in 7,9-*O-*Ac was seen in either ΔSIAE and ΔSIAE+CasD1 cell lines by HPLC analysis. Both ΔSIAE+CasD1 lines had altered growth rates compared to WT cells: HEK-293 ΔSIAE+CasD1 had delayed growth rates, while A549 ΔSIAE+CasD1 had increased growth rates. This suggests that dysregulation of SIAE and CasD1 by gene manipulation affects cell metabolism and growth in a cell-type-specific manner, possibly through the buildup of glycoproteins in the Golgi or through dysregulation of the sialic acid recycling pathway. These effects on cell growth could have implications for cancer and organismal development, since these variant Sia are involved in both these processes (15–17, 20–23, 49). Further research is needed to determine how 7,9-*O*- and 9-*O*-Ac expression is regulated, how they are transported within the ER and Golgi bodies, and what roles they play in cell growth. Of particular interest will be disentangling the individual regulation of 7,9-*O-*Ac expression compared to 9-*O-*Ac, since there do seem to be differences in how they are regulated in cells in the present study versus previously reported expression in animal tissues (10, 11).

Studies with two strains of IAV and two strains of IBV showed no differences in infection efficiency in WT HEK-293 or MDCK-NBL2 cells compared to their ΔCasD1 or CasD1-OX variants. This is not surprising, since >95% of the Sia is unacetylated Neu5Ac, which can be utilized by IAV and IBV as a receptor for binding and entry. Although the low levels present on cells were not enough to affect either IAV and IBV infection, we did determine that higher levels, such as those found on mouse erythrocytes (∼45% *O-*Ac), were able to block IAV HA binding and decrease IAV NA cleavage efficiency in a strain-dependent manner. It is possible, therefore, that if higher levels are present on secreted proteins in mucus, they could inhibit infection prior to the virus reaching the cell surface. Further analysis of mucus *O-*acetylation in IAV hosts would be necessary to determine whether 7,9-*O-* and 9-*O-*Ac could contribute to the barrier function of mucus in respiratory tissues (41).

The 1 to 2% of 9-*O*-Ac on the surfaces of WT MDCK-NBL2 cells was sufficient for ICV and IDV binding and entry, and thus cell susceptibility was lost when CasD1 was inactivated. Interestingly, there does still seem to be a necessary minimum threshold of 9-*O*-Ac presence needed for some ICV and IDV strains to infect cells, since the C/Ann Arbor and C/Victoria strains were able to infect some MDCK-CasD1 OX cells at a lower level than WT MDCK cells, whereas the C/Taylor strain was not. Similarly, IDV also showed lower levels of infectivity in MDCK-CasD1 OX cells compared to WT MDCK cells. It is likely that similar results would be seen for other viruses that use these modified Sia as a receptor, including human coronaviruses OC43 and HKU1 (50).

In summary, we have shown that these modifications are present in different cell lines sourced from different species of animals, but they make up a small minority of the total Sia present. In addition, these modifications vary considerably in their localization and have an inherent heterogeneity within cell populations. While the presence of both 7,9-*O*- and 9-*O*-Ac were dependent on the activity of CasD1, the relative proportions, levels of expression, and localization appear to be controlled by more complex mechanisms than simply the expression of CasD1 and SIAE. How this regulated expression affects cell homeostasis is unknown, but it is likely relevant during development, immune responses, and in cancers that show dysregulation of 7,9-*O*- and 9-*O*-Ac expression. For viruses such as ICV and IDV that rely on 9-*O*-Ac for infection, the low levels seen on cell surfaces are sufficient for infection. We found that 7,9-*O*- and 9-*O*-Ac do have inhibitory activity on HA binding and NA cleavage when present at higher levels. Although these modifications are present on cell surfaces at levels too low to affect IAV and IBV infection, they are expressed at much higher levels in mucosal tissues and in secreted mucus proteins of many animals, which may provide a more effective barrier (10, 11, 51–53). We are currently examining these processes for secreted mucus and other sources in different animals.

## MATERIALS AND METHODS

### Cells and viruses.

HEK-293, A549, MDCK-NBL2, MDCK type I, and MDCK type II cells were grown in Dulbecco modified Eagle medium (DMEM) with 5% fetal bovine serum and 50 μg/ml gentamicin. HEK-293, A549, and MDCK-NBL2 cells were obtained from the American Type Culture Collection (ATCC). MDCK type I and type II cells were gifts from William Young (University of Kentucky) (35, 37, 54, 55). SiNEC and SiTEC cells were gifts from Stacey Shultz-Cherry (St. Jude Children’s Hospital). IAV strains pH1N1 (A/California/04/2009) and Victoria H3N2 (A/Victoria/361/2011) were rescued from reverse genetics plasmids using established protocols (56, 57). Rescued viruses were grown to low passage on MDCK-NBL2 cells using infection media containing DMEM, 0.03% bovine serum albumin (BSA), and 1 μg/ml TPCK (tolylsulfonyl phenylalanyl chloromethyl ketone)-treated trypsin. IBV strains B/Colorado/06/2017 and B/Memphis/1/2018, and IDV strains D/bovine/MS/C00020N/2014 and D/swine/OK/1334/2011 were gifts from Richard Webby (St. Jude Children’s Hospital) and were grown in infection media containing DMEM, 0.03% BSA, and 1 μg/ml TPCK-treated trypsin. ICV strains C/Ann Arbor/1/50 and C/Taylor/1233/1947 were gifts from Andrew Pekosz (Johns Hopkins University), and ICV strain C/Victoria/1/2011 was a gift from Richard Webby (St. Jude Children’s Hospital). All ICV strains were grown on MDCK-NBL2 cells using infection media containing DMEM, 0.03% BSA, and 5 μg/ml TPCK-treated trypsin.

### Immunofluorescence microscopy and flow cytometry.

Cells were stained using probes derived from viral hemagglutinin-esterase proteins fused to human IgG1 Fc (HE-Fc). The porcine torovirus strain 4 HE-Fc (PToV HE-Fc) primarily recognizes 9-*O*-Ac, and the bovine coronavirus Mebus strain HE-Fc (BCoV HE-Fc) recognizes 7,9-*O*-Ac and shows low levels of binding to 9-*O*-Ac (10, 11). For immunofluorescence microscopy, the cells were seeded onto glass coverslips and incubated overnight at 37°C and 5% CO_2_. Coverslips were fixed in 4% paraformaldehyde (PFA) for 15 min. Coverslips were incubated with Carbo-Free blocking solution (Vector Laboratories) for 1 h at room temperature, with optional permeabilization with 0.001% Tween 20. For staining, HE-Fc probes were precomplexed with Alexa 488-labeled anti-human IgG antibody for 1 h at 4°C and then diluted in Carbo-Free blocking solution to a final concentration of 5 μg/ml HE-Fc and a 1:500 dilution of secondary antibody. Cells were stained with HE-Fc/anti-IgG complex for 1 h at room temperature. Coverslips were mounted using Prolong Antifade-Gold with DAPI (4′,6′-diamidino-2-phenylindole; Invitrogen). Cell were imaged using a Nikon TE300 fluorescence microscope. For flow cytometry, cells were seeded onto nonadherent cell culture dishes and incubated overnight at 37°C and 5% CO_2_. Cells were collected using ice-cold phosphate-buffered saline (PBS; HEK-293 and A549 cells) or Accutase (Sigma, MDCK cells) to retain surface glycans and then fixed in 4% PFA for 15 min. The cells were blocked as described above. HE-Fc probes were prepared as described above with final concentrations of 5 μg/ml HE-Fc probe and 1:1,200 of anti-IgG. A Guava EasyCyte Plus flow cytometer (EMD Millipore, Billerica, MA) was used to collect data, followed by analysis using FlowJo software (TreeStar, Ashland, OR). Statistical analyses were performed using GraphPad Prism software (v8).

### Cell line mutations and characterization.

Two methods for utilizing CRISPR-Cas9 were used. For A549 and MDCK cells, paired Cas9 plasmids (PX459; Addgene plasmid 62988) targeted adjacent sites in early exons of CasD1, as diagrammed in Fig. 4A. Plasmids were transfected using TransIT-X2 (Mirus Bio LLC) (8). For HEK-293 cells, nickase Cas9 plasmids (PX462; Addgene plasmid 62987) were used instead. Transfected cells were selected with puromycin, and single cell clones were screened with PToV-P4 HE-Fc to identify nonstaining variants. Edited sequences were confirmed by PCR amplification of the targeted regions and sequencing the PCR product for each allele. Knockout cell lines were used to prepare overexpression cell lines by transfection of a pcDNA3.1(–) plasmid expressing the complete human CasD1 cDNA open reading frame synthesized by Bio Basic (Markham, Ontario, Canada). Transfected cells were selected with G418, and single-cell clones were screened by staining with PToV-P4 HE-Fc to identify 9-*O-*Ac positive cell lines. Editing of the SIAE gene followed a protocol for CRISPR-Cas9 similar to that described above, and the gene regions targeted are shown in Fig. 5A. After transfection and selection, cells were cloned, and single-cell clones were screened by direct PCR amplification of the target gene region and analysis of the PCR product size for the edited form of both alleles. Full sequencing of each allele and qPCR were performed to confirm deletion of the gene.

### Quantification of Sia variants.

The Sia composition of cells were determined by incubating with 2 M acetic acid at 80°C for 3 h, filtration through a Microcon 10-kDa centrifugal filter (Millipore), and drying in a SpeedVac vacuum concentrator. Released Sia were derivatized with DMB (Sigma-Aldrich) for 2.5 h at 50°C (39). HPLC analysis was performed using a Dionex UltiMate 3000 system with an Acclaim C_18_ column (Thermo Fisher) under isocratic elution in 7% methanol, 7% acetonitrile, and 86% water. Sia standards included bovine submaxillary mucin and commercial standards for Neu5Ac and Neu5Gc (Sigma-Aldrich). Statistical analyses were performed in GraphPad Prism software (v8).

### Characterization of A549 conditioned media.

Conditioned medium from A549 cells was prepared by washing a fully confluent flask of cells to remove any serum and allowing the cells to grow in serum-free medium for 5 to 7 days. The conditioned medium was collected, dialyzed with 3 volumes of PBS, and concentrated using a 30-kDa centrifugal filter (Pall Corporation). The protein concentration was determined by using a Qubit 4 fluorometer (Invitrogen). To determine Muc5B presence, 2-fold dilutions of conditioned media were compared to purified human Muc5B using a 8% SDS-PAGE gel and probed with an anti-human Muc5B antibody (both purified protein and antibody were gifts from Stefan Ruhl, University of Buffalo). Conditioned medium was analyzed for total Sia using the HPLC methods listed above.

### qPCR of SIAE and CasD1 expression.

RNA from cells was extracted by using EZNA total RNA kit I (Omega Bio-Tek), and cDNA was synthesized using the SuperScript II reverse transcriptase (Invitrogen) standard protocol with oligo(dT)_12-18_ primers (Invitrogen). CasD1- and SIAE-specific primers were designed using Geneious (Biomatters, Ltd.). For CasD1, adequate primers could not be targeted around the CRISPR-Cas9 edit site due to the high G/C content. qPCR was performed on a Applied Biosystems StepOnePlus real-time PCR system using Fast SYBR green (Bio-Rad). Data were analyzed using StepOne software (Applied Biosystems, v2.1) and GraphPad Prism statistical analysis software.

### Virus infection assays.

For infection in HEK-293 cells with IAV, the cells were seeded on coverslips and inoculated with a multiplicity of infection (MOI) of 0.1. Coverslips of inoculated cells were fixed at 24 h, stained for virus using an anti-NP antibody, and costained with DAPI. The percentages of infected cells were determined by imaging coverslips and counting the infected cells per field. Statistical analyses were performed in GraphPad Prism software. For infection in MDCK cells with the influenza A, B, C, and D viruses, cells were seeded into 96-well plates and inoculated for 48 h. The cells were then fixed and stained with a mouse anti-influenza A NP antibody, a mouse anti-influenza B NP antibody (Abcam), a polyclonal mouse anti-influenza C antibody (a gift from Peter Palese, Icahn School of Medicine at Mount Sinai), or a polyclonal rabbit anti-influenza D antibody (a gift from Feng Li, South Dakota State University). The cells were imaged using a Nikon TE300 fluorescence microscope. Images were analyzed for relative infected cell counts using ImageJ (NIH and LOCI, University of Wisconsin).

### Mouse erythrocyte hemagglutination and lectin staining.

Mouse erythrocytes were collected from C57B/6 mice by euthanizing the mice and immediately collecting blood by heart puncture into Alsever’s solution. Erythrocytes were washed in PBS three times and diluted to 5% (vol/vol) in PBS. Then, 5% of the mouse erythrocytes were left untreated or treated with 30 μg/ml BCoV HE-Fc for 18 h at 37°C, followed by dilution to 0.75% (vol/vol) in PBS for the HA assays. Briefly, HA assays were performed by 2-fold serially diluting virus in duplicate per treatment in a V-bottom 96-well plate. Treated or untreated mouse erythrocytes were added to virus and allowed to hemagglutinate at 4°C to prevent NA cleavage. To determine Sia linkage type, 5% washed erythrocytes were blocked for 1 h using 1× Carbo Free blocking buffer (Vector Laboratories) and then stained for 1 h with fluoroscein-labeled plant lectins SNA, which binds α2,6-linked Sia, and MAA I, which binds α2,3-linked Sia (Vector Laboratories). Stained erythrocytes were then analyzed using flow cytometry as described above.

### Generation of NA VLPs and NA cleavage assay.

NA sequences were obtained from GenBank (N1, ACP44181; N2, AGC70842). Sequences were tagged, codon optimized, and ordered through Biomatik in the PcDNA3.1(+) vector. To produce VLPs, HEK-293T cells were seeded in 15-cm plates and transfected when 80% confluent. The cells were transfected with 4 μl of polyethylenimine (Polysciences, catalog no. 23966-2) at 1 mg/ml for every 1 μg of plasmid DNA stock of in 9 ml of Opti-MEM. At 8 h posttransfection, 6 ml of prewarmed Opti-MEM was added. The supernatant was collected at 72 h posttransfection and purified using ultracentrifugation (110,000 × *g*, 1.5 h, 4°C) through a 20% sucrose cushion. The pellet was resuspended in PBS and stored at 4°C. To determine NA cleavage on mouse erythrocytes, 5% (vol/vol) mouse erythrocytes in PBS were treated with 1:100 NA VLPs for 4 h at 37°C. Free Sia were collected and prepared for HPLC analysis as described above.
