# Supplementary figures and images for: Expression of 9-O- and 7,9-O-Acetyl Modified Sialic Acid in Cells and Their Effects on Influenza Viruses
Source: mBio. 2019 Dec 3;10(6):e02490-19. doi: 10.1128/mBio.02490-19 (PMC6890989; doi:10.1128/mBio.02490-19)

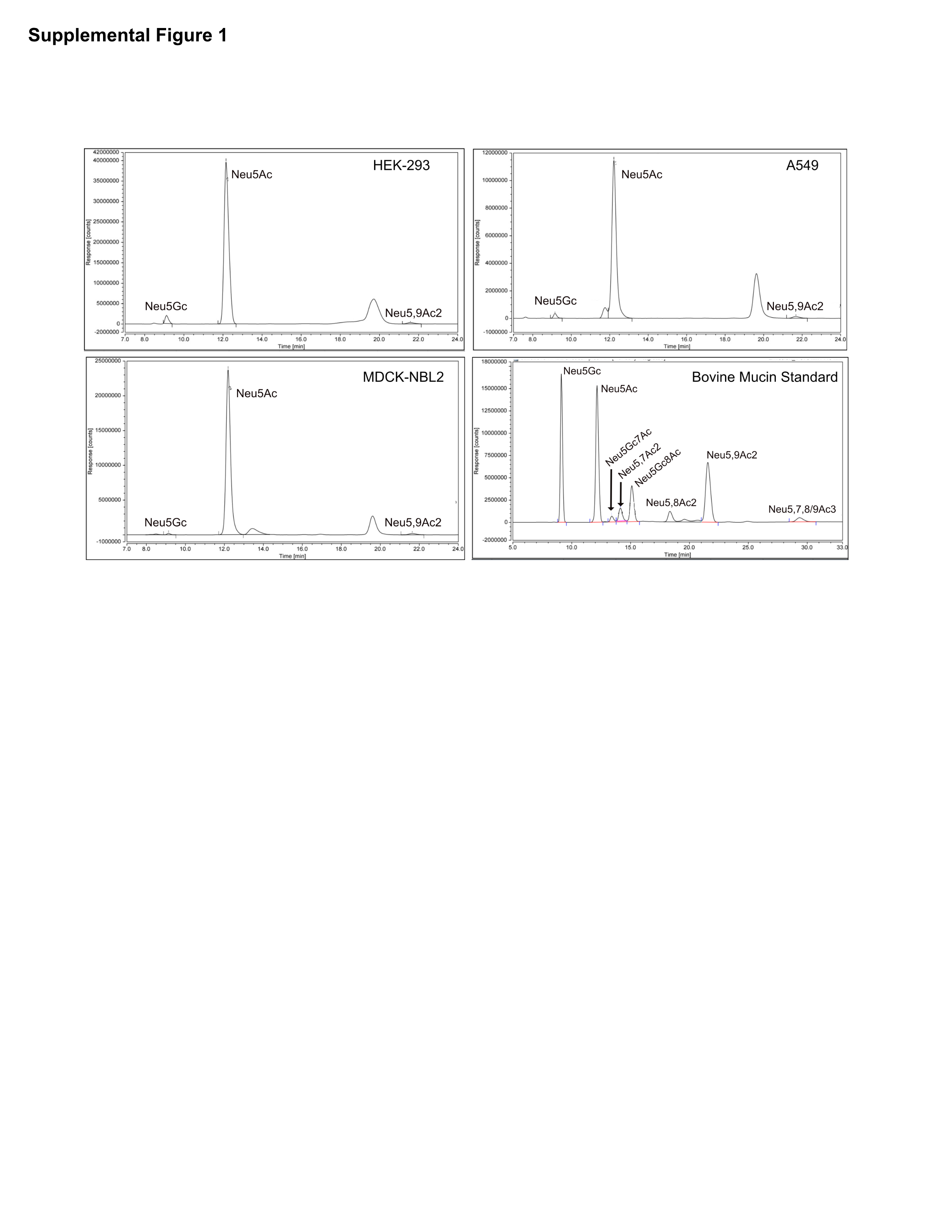

Supplement: FIG S1 [file mBio.02490-19-sf001.tif]

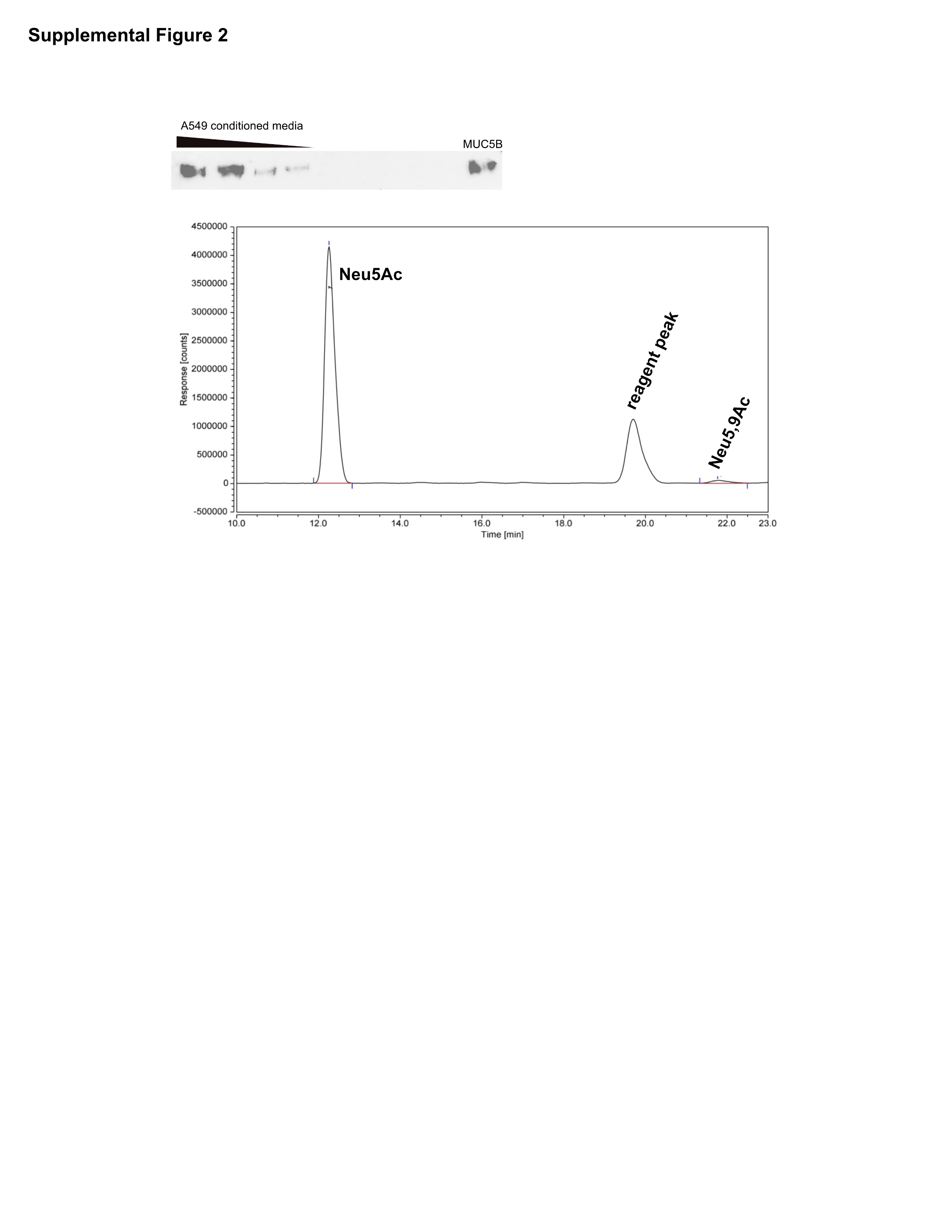

Supplement: FIG S2 [file mBio.02490-19-sf002.tif]

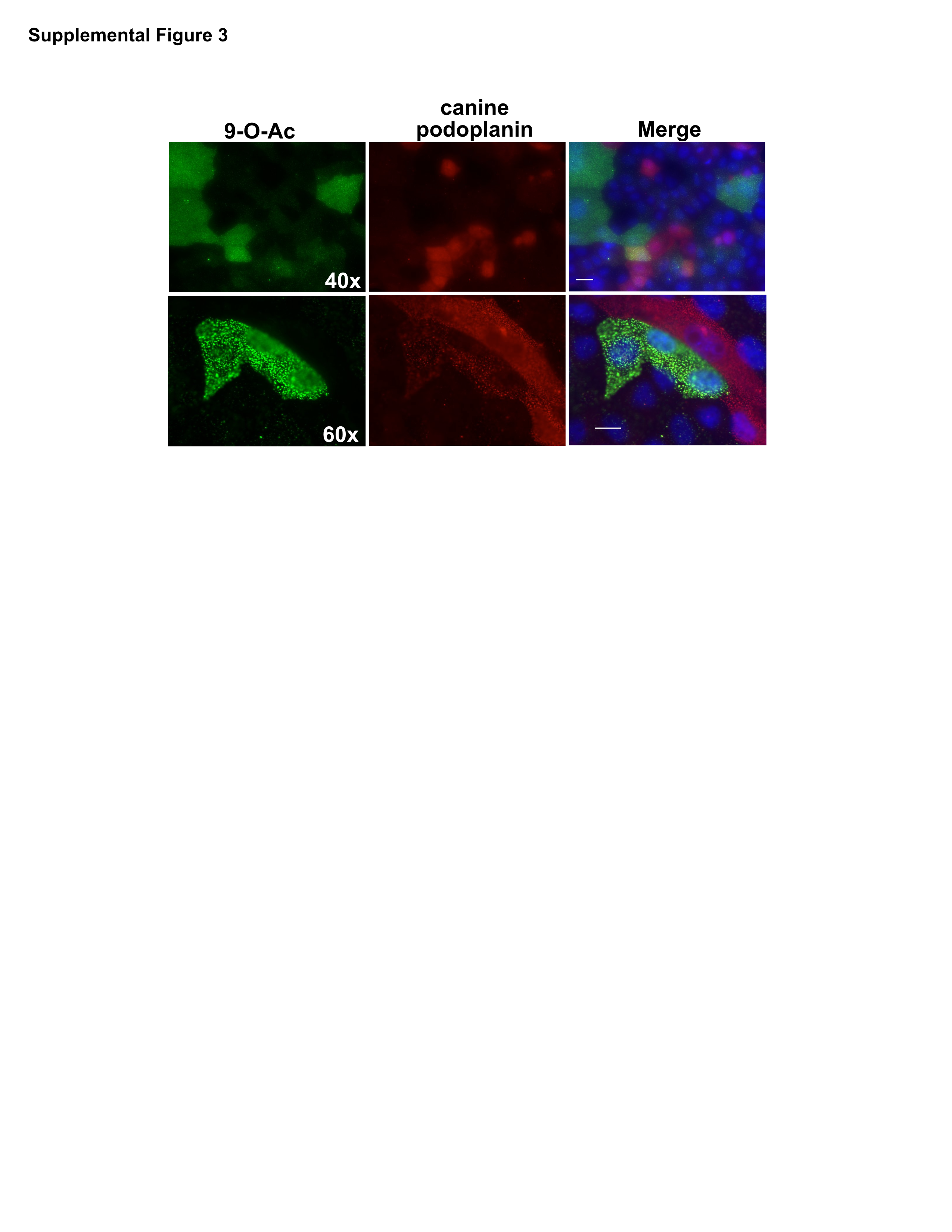

Supplement: FIG S3 [file mBio.02490-19-sf003.tif]
